# Exploring the Effects of Novel Food Processing Methods on Food Proteins: A Review

**DOI:** 10.1002/fsn3.70373

**Published:** 2025-06-11

**Authors:** Shuai Wei, Anand Kumar, Gebremichael Gebremedhin Hailu, Sun‐Il Choi, Ok‐Hwan Lee, Ramachandran Chelliah, Deog‐Hwan Oh, Shucheng Liu

**Affiliations:** ^1^ College of Food Science and Technology Guangdong Ocean University, Guangdong Provincial Key Laboratory of Aquatic Products Processing and Safety, Guangdong Province Engineering Laboratory for Marine Biological Products, Guangdong Provincial Engineering Technology Research Center of Seafood, Key Laboratory of Advanced Processing of Aquatic Product of Guangdong Higher Education Institution Zhanjiang China; ^2^ Department of Food Engineering Oda Bultum University Chiro Ethiopia; ^3^ Department of Food Science and Biotechnology College of Agriculture and Life Sciences, Kangwon National University Chuncheon Republic of Korea; ^4^ Collaborative Innovation Centre of Seafood Deep Processing Dalian Polytechnic University Dalian China

**Keywords:** functional properties, novel food processing, protein structure

## Abstract

Novel food processing technologies, such as ohmic heating, ultrasonic heating, cold plasma, high‐pressure processing (HPP), pulsed electric fields (PEFs), and enzymatic hydrolysis, have the ability to extend shelf life, improve quality, regulate the freshness of food products, and have diverse effects on food components, that is, protein, fat, and carbohydrates. Understanding the overall effects and mechanisms of these methods on food proteins from a multidimensional perspective is a core foundation for driving further innovations and advancements related to food protein modifications and their applications in the food industry. Therefore, this review aims to explore novel food processing technologies and their impacts on the particle size, structure, solubility, emulsion stability, foaming properties, and bioactivity of food protein, which affect the nutritional and functional properties of foods. Recent studies have shown that all these technologies have a significant effect on protein structure, solubility, functionality, and bioactivity. For instance, HPP primarily affects the particle size, secondary structure, and coagulation properties. PEF has been shown to enhance protein solubility and modify protein structure. Enzymatic hydrolysis breaks down proteins, improving their texture, proteolytic activity, degree of hydrolysis, and solubility. Processing‐induced changes in protein properties significantly enhance the overall qualities of the final food products. While novel food processing methods show promise for enhancing food proteins, they also have several drawbacks. To reduce these negative effects, combining different processing techniques may offer a solution, and computer‐based tools can help simulate, optimize, control, and validate these processes.

## Graphical abstract

This review provides a comprehensive overview of the effects of novel food processing methods on food proteins and their implications for protein‐based food products. Furthermore, the review highlights future perspectives on novel food processing and food protein modifications.

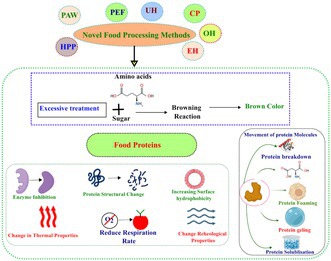

## 1 Introduction

Proteins, as sources of essential amino acids and fundamental components of the human diet, play a crucial role in human health (Rezzi et al. 2024) and are obtained from several sources, including microorganisms, plants, animals, and fungi. Plants provide the majority of the protein in diets worldwide, accounting for more than 60% of the total protein supply. Animal‐based foods such as fish, poultry, dairy, and meat meet the remaining protein requirements (FAO 2015). In addition to their nutritional, bioactive, and physiological benefits, food proteins possess functional qualities, including solubility, water retention, fat absorption, emulsification, foaming, and gelation. The characteristics of food proteins render them versatile and valuable components in diverse product creation, formulation, and many applications. Notwithstanding their appealing attributes, suitable processing may be required to modify, enhance, or customize these molecular properties for particular food uses. Because of their sensitivity to applied treatments, the structure and functionality of proteins can change during food processing (Sagis and Yang 2022).

The food industry is striving to provide healthy, preservative‐free, high‐quality, safe food products to its customers. Several novel technologies have been developed in the last few decades to ensure food quality and safety. Radiofrequency heating, ohmic heating, ultrasonication, irradiation, Ozen and CO_2_ processing, high‐pressure processing, plasma‐activated water, fermentation, and enzymatic hydrolysis are among these methods (Ling et al. 2020). Compared with traditional procedures, these innovative processes better preserve organoleptic and nutritional properties while ensuring food safety. For innovative food processing processes, the whole processing time is short, and energy usage is minimal (Huang et al. 2024). Research has focused on understanding the effects of these technologies on food structure, color, flavor, health‐related components, and allergens (Morales‐de la Peña et al. 2019). Furthermore, the impacts of processing on protein structure and allergenicity are also of interest and have been investigated.

Every novel processing method has its own impact on the functional, rheological, structural, microbiological, and sensory properties of food. Changes in the properties of proteins as a result of processing highly contribute to changes in the functional and rheological properties of food (Wu et al. 2020). Hence, proteins are composed of long chains of amino acids, and novel food processing techniques have complex and wide‐ranging effects. For instance, structural changes, that is, secondary, tertiary, and quaternary structures, as a result of various food processing treatments, significantly affect functional properties such as the solubility, emulsifying, and foaming capacity (Turker and Isleroglu 2024), and gelation (Sinthusamran et al. 2023) properties of food proteins.

Exploring the effects of novel food processing methods on food proteins is a foundation for their application in food processing and protein modification. From a nutritional point of view, structural changes in proteins caused by treatments may enhance the digestibility of food proteins (Chian et al. 2019), resulting in improved amino acid absorption (Cosson et al. 2022). Despite their positive impacts, prolonged and unoptimized treatments might lead to protein denaturation and loss of essential amino acids (Zou et al. 2024). In addition, modifications in the functional properties of proteins significantly affect the texture, appearance, flavor, and rheological properties of food products. Nemati and Guimarães (2024) reported improvements in the appearance, color, and taste of Mozzarella cheese treated with plasma‐activated water (PAW), that is, a promising novel food processing method, as a result of improving the gelation properties of its protein. Improvements in the firmness and consistency of products such as yogurt have been reported as a result of improvements in protein gelation properties (Gantumur et al. 2024).

Moreover, modifications of food protein properties through novel food processing methods provide an opportunity for product innovation and enhancement of existing product properties, as research into improving the functional, physicochemical, and rheological properties of food proteins from various sources (Baskıncı and Gul 2023; Hu, Yu, et al. 2023), as well as the development of meat analogs from modified proteins (Jiang et al. 2024), is gaining interest. Thus, the authors believe that exploring and organizing recent research on the effects of various novel food‐processing methods on food protein and highlighting potential future directions will help researchers find gaps and advance this sector.

Although a previous review presented a valuable overview of the effects of some biological and conventional methods on the structure, functionality, and bioactivity of food proteins (Wu et al. 2020), this review summarized the most recent novel food processing methods and their impacts on the structure, functionality, and bioactivity of proteins originating from different sources on the basis of recent studies and findings. The current review is also designed to address at least the following questions: (i) What are the mechanisms by which novel food processing methods modify food proteins? (ii) How do various novel food‐processing methods compare in terms of their effects on food proteins? (iii) How do novel food processing methods affect the structure of food proteins? (iv) How do the effects of novel food processing methods on food proteins affect final food product quality? (v) Can novel food processing methods be optimized to achieve specific protein‐related outcomes in food products? (vi) What are the potential applications of modified food proteins resulting from novel processing methods in the food industry? Furthermore, this review provides future perspectives on novel food processing methods and their possible impacts on food proteins.

## 2 Ohmic Heating

### 2.1 General Descriptions and Working Principles

**FIGURE 1:**
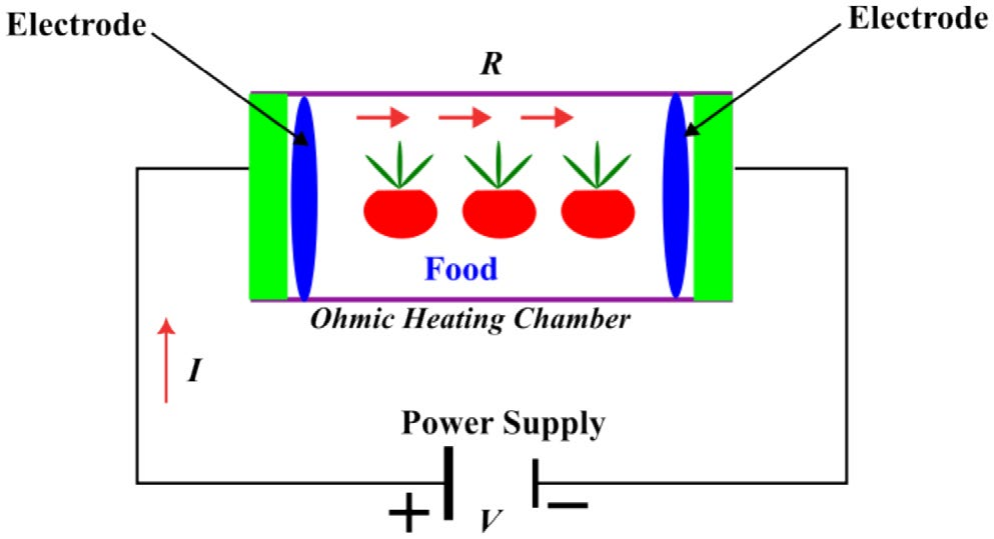
Working principles of ohmic heating (dos Santos et al. 2024).

Ohmic heating is an innovative approach frequently employed for thermal processing. A food product is placed between two electrodes, functioning as an electrical resistor, while an alternating electric current is transmitted across the circuit. Heat is generated throughout food as a result of electrical resistance, and its generation occurs throughout the volume. The electrical energy is turned into heat, resulting in an increase in temperature. In principle, food is made part of an electrical circuit. Compared with conventional heating, ohmic heating provides rapid heating of foods (Morya et al. 2022). A schematic representation of ohmic heating is presented in Figure 1.

Although it is well known for its uniform heating, excellent energy efficiency, and preservation of nutritional and sensory properties, it has several drawbacks (Aurina and Sari 2022), including fouling deposits, high initial cost, and limited awareness of the technology. It is less effective with nonconductive foods, particularly those high in fats and oils. The high initial cost and lack of broad research make system control difficult (Indiarto and Rezaharsamto 2020). Several factors influence the ohmic heating of foods, primarily electrical conductivity and field strength, which can change during heating due to several factors, such as starch transition, fat melting, and cell structure changes. Food composition, particularly the presence of salt and suspended particles, has an impact on the ohmic heating efficiency (Jan et al. 2021).

### 2.2 Impact of Ohmic Heating on Food Proteins

Safari et al. (2025) investigated the effects of ohmic thawing variables and freezing methods on Turkish meat and found immersion freezing during ohmic heating decreased protein quality, and significant denaturation was obtained during storage for 90 days at −18°C. The snap‐freezing approach resulted in the lowest protein solubility due to reduced structural damage. The snap‐freezing process generates small ice crystals, hence reducing protein degradation and enhancing protein retention in the product. The maximum protein solubility was obtained the freezing procedure at temperatures of −70°C and −20°C, with a voltage gradient of 15 V/cm. Freezing at −20°C led to the production of larger ice crystals, causing increased protein degradation and subsequent conversion into smaller peptides. Increasing the amount of peptides increases protein solubility, leads to increased protein release after thawing, and decreases the nutritional value of meat (Fattahi and Zamindar 2021).

Ohmic heating affects fat content and protein structure, and their interactions strongly affect milk viscosity and sensory properties (Sun et al. 2024). Another study reported an enhancement in the water and oil holding capacity, emulsifying properties, and foaming characteristics of sesame protein isolate following ohmic heating treatment (Saeedabad et al. 2024). However, the particle size and turbidity increased after ohmic heating. Balthazar et al. (2024) reported that ohmic heating improved the bioactivity of sheep milk by facilitating proteolysis, which produces peptides with biological activities such as antimicrobial, antioxidant, antihypertensive, antithrombotic, and immunomodulatory properties. Ohmic heating can trigger the release of enzymes responsible for protein cleavage, including casein fractions and whey protein, leading to increased bioactive substances (Salari and Jafari 2020).

Ohmic heating increased the density, average size, and percentage of bread pores due to protein network development and polymerization (Sutrisno et al. 2025). These findings demonstrated that bread made via ohmic heating had increased hardness, springiness, and chewiness, which are directly related to protein denaturation and hydration. Chen et al. (2023) reported a slight increase in the ketone content of peanut protein isolate during ohmic heating, which could be attributed to the oxidation of the alkanoic acid chain in the protein. Ferreira et al. (2021) used ohmic heating for whey processing and found a decrease in consistency due to the production of a nonuniform gel, as well as an increase in viscosity due to protein denaturation. A summary of some examples of the effects of ohmic heating on food proteins is provided in Table 1.

**TABLE 1:** Typical examples of the effects of ohmic heating on food proteins.

Protein sources	Ohmic heating conditions	Major observations	References
Flavored milk	– Electric field strengths (5.22, 6.96, 8.7, and 10.43 V/cm)	– Compared to conventional pasteurization (PAST, 72°C/15 s), ohmic heating resulted lowest consistency, implying weaker and thinner protein network structure – Ohmic heating at 6.96 and 8.7 V/cm showed the highest biological activity, suggesting bioactive peptide generation due to milk protein kinetics	Rocha et al. (2022)
Japanese eel	– Case 1 (Ohmic heating + water bath heating) (OH + WH) – Case 2 (Ohmic heating + air thermostatic heating) (OH + AH)	– Ohmic heating altered charge state on the surface of the protein → promoting protein aggregation. – Higher amino acid was recorded for combined OH + WH treatments – Following OH treatment, β‐sheets and unordered structures ↑, α‐helixes ↓	Li, Deng et al. (2024)
Sheep milk	– Electric field strengths (3.33–8.33 V/cm)	– Ohmic heating accelerated proteolysis breakdown of milk protein → bioactivity improvement	Balthazar et al. (2024)
Turkey meat	– Voltage gradient (10, 15, and 20 V/cm) – Freezing method (freezing samples with liquid nitrogen at −210°C, −70°C, and −20°C)	– 15 V/cm voltage gradient and freezing at −70°C resulted highest solubility – Prolonged treatment resulted a decrease in pH, indicating protein denaturation and the formation of H^+^	Safari et al. (2025)
Pea	– Electric field strength (25 V/cm) – Electrical frequency (20 kHz) – Treatment temperature (90°C–150°C)	– Ohmic treatment at 150°C increased protein solubility by 237% – Samples treated ohmic heating exhibited a higher content of β‐sheet and random coil – Hydrodynamic diameter (Zavg) of particles increased until 120°C treatment temperature	Avelar et al. (2024)
Sesame	– Frequency (50 Hz) – Voltage (0–220 V) – Constant temperature of 85°C	– Employing ohmic heating resulted in positive zeta potentials ranging from 42.7 to 108 mV – Relatively lower in total and free sulfhydryl content than the untreated samples – A significant increase in solubility from 4.1% to 47.8% was observed following ohmic heating. – Water holding capacity (292%–360%) and foaming capacity (11.6%–13.86%) was significantly increased	Saeedabad et al. (2024)

## 3 Ultrasound Heating

### 3.1 Working Principles and Frequency Ranges

**FIGURE 2:**
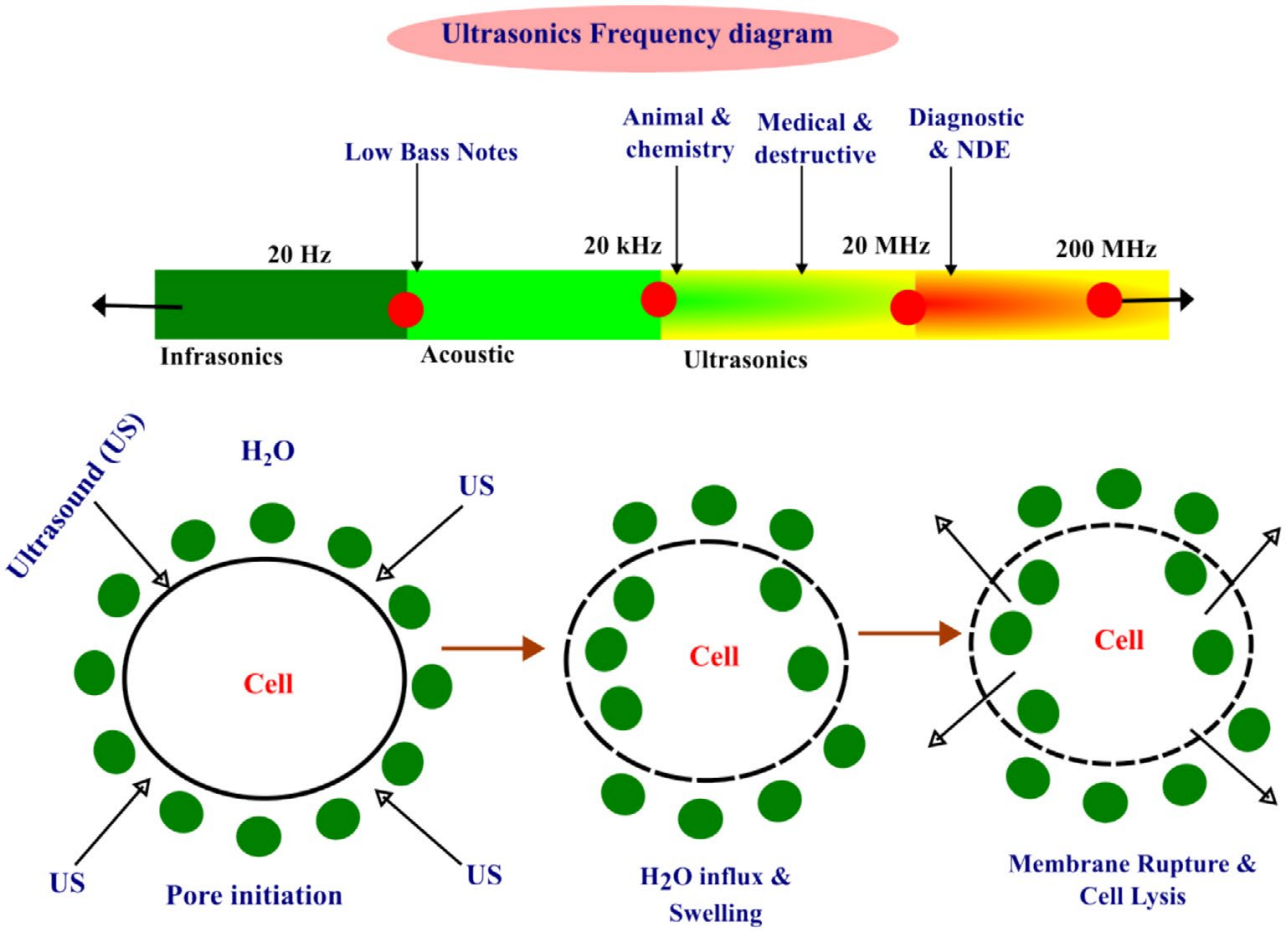
Ultrasound frequency ranges and mechanisms (Singla and Sit 2021).

Ultrasonic processing is a nonthermal processing method that can inactivate microorganisms and enzymes through cavitation at high‐frequency sound waves. Ultrasound treatment essentially results in a sound wave with a frequency greater than 20 kHz (Figure 2), which can be classified as low frequency (20–100 kHz) or high frequency (100–1000 kHz) (Singla and Sit 2021). Ultrasound technology has transformed the food‐processing sector through its extensive application in several processes, acting as a sustainable and cost‐effective option. This nondestructive method provides numerous benefits, including expedited operations, increased efficiency, reduction of procedural steps, superior product quality, preservation of product attributes (texture, nutritional content, organoleptic features), and extended shelf life (Bhargava et al. 2021).

Ultrasound technology in food processing provides multiple benefits, such as increased mixing, accelerated energy and mass transfer, decreased processing duration, and enhanced product quality. The cavitation phenomenon caused by high‐intensity ultrasound can result in localized elevated temperatures and pressures, impacting the quality of numerous foods (Jadhav et al. 2023). Despite its efficiency in eliminating pathogenic bacteria, it has several drawbacks, including high starting costs, the possible production of free radicals, sensory property loss with longer processing times, and the possibility of physical and chemical changes in food (Rathnakumar et al. 2024).

### 3.2 Impact of Ultrasound Heating on Food Proteins

In recent years, ultrasound treatment has been applied for food processing for different purposes, that is, emulsification, dispersion, preservation, stabilization, dissolution, and crystallization. Ultrasound treatment, a promising processing technique, significantly enhances the physicochemical, functional, and rheological properties of food proteins. The reports on the effects of ultrasonic treatments on food protein are summarized in Table 2. Ultrasonic treatment enhances protein digestibility and reduces the particle size of meat pure protein (Luo et al. 2021). Ultrasound can result in alterations in protein structure, leading to the exposure of enzyme cleavage sites, which facilitates protein breakdown and enhances protein digestibility (Qian et al. 2023). Pacheco et al. (2023) investigated the effects of ultrasonic treatment on pumpkin seed protein concentrate hydrolysis and reported that ultrasonic treatment resulted in an increase in the proteolysis rate, soluble peptide content, and biological activities of proteins. The production of smaller peptides and exposure of polar and ionizable groups (amino and carboxyl groups) increase the hydrophilicity of the hydrolysates, enhancing protein solubility (Devaki and Ghosh 2024). Furthermore, (Sinthusamran et al. 2023) reported that ultrasonic treatment altered protein patterns, increased water solubility, resulted in protein aggregation and structural changes, improved gelling properties, and caused muscle proteins to release myoglobin, resulting in a reduction in the myoglobin concentration of yellow stripe scad protein. Other authors also reported the effects of ultrasonic treatment on the physicochemical, functional, and bioactivities of cress seed protein (Turker and Isleroglu 2024), milk protein (Zhao et al. 2024), egg protein (Zhang, Liu, et al. 2024), fish protein (Rajasekaran et al. 2023), and porcine myofibrillar protein (Yu et al. 2024).

**TABLE 2:** Effects of ultrasound treatments on different food proteins.

Protein sources	Variables and effects on the food protein	References
Meat puree	– Ultrasound (20 kHz and, 200, 400, and 600 W), Time (15, 30, 45) – ↑Ultrasound power and time, protein digestibility ↑, particle size ↓	Luo et al. (2021)
Pumpkin seed	– Ultrasound (40 kHz, 23.8 W/L), Temperature (25°C–60°C), and Time (5, 15, 30, 60, 90, 120, 180, 240, and 300 min) – Ultrasound‐assisted hydrolysis resulted up to 280% increase in the proteolysis rate – Higher (24%) concentration of soluble peptides (at 120 min, 60°C) achieved after ultrasound treatment – Improved in vitro antioxidant activity (biological activity)	Pacheco et al. (2023)
Yellow stripe scad	– Ultrasound (45 kHz, 250 W) and Time (10 min) – Muscle protein release myoglobin → Myoglobin content ↓ – Ultrasound‐assisted washing increased myofibrillar proteins → protein solubility ↓, water‐holding capacity ↑ – Alterations of protein pattern (concentrates myofibrillar proteins, such as MHC/myosin heavy chain and AC/Actin, and reducing sarcoplasmic proteins) – Protein aggregation and conformational change → gelling properties ↑	Sinthusamran et al. (2023)
Cress seeds	– Ultrasound (20 kHz, 500 W), pH (3, 4, and 5), Temperature (30, 40, and 50), and Time (1, 2.5, and 4 h) – Increased foaming capacity (62%) and water holding capacity of the protein – Higher emulsion stability (20.2 min) of the protein – Notably better solubility (7.90 g/L) at 12 pH – Ultrasonication process did not significantly alter the protein structure	Turker and Isleroglu (2024)
Pumpkin seed	– Ultrasound (20 kHz, 500 W, and 25% amplitude) and Time (5, 10, 20, and 30 min) – Higher solubility at 20 min sonication time – Improved emulsifying properties, but ↑ sonication time → emulsifying properties ↓ – Sonication decreased particle size – Resulted lower turbidity – Improved antioxidant activity (DPPH scavenging activity)	Habib et al. (2025)
Milk	– Ultrasound power (150, 300, and 500 W) and Ultrasonication time (5, 10, and 15 min) – Ultrasound changed the secondary and tertiary structure of the protein – Particle size distribution decreased and uniform particle size distribution of the sample	Zhao et al. (2024)
Sea bass	– Ultrasonic powers (100, 300, and 500 W) and Ultrasonication time (3 min) – Increased protease activity – Higher level of protein degradation	Bai et al. (2023)
Fish	– Ultrasound (20 kHz, 750 W) and Ultrasonication time (5, 10, and 15 min), Amplitudes (40% and 60%) – Altered protein pattern (decrease in MHC/myosin heavy chain band inanities) – Increased solubility by > 19.26% – Increased the surface hydrophobicity – Enhanced emulsifying properties	Rajasekaran et al. (2023)
Egg	– Ultrasonic power (400 W) and Ultrasonication time (0, 4, 8, 12, 16, and 20 min) – Free amino acid content decreased – Increased the solubility and surface hydrophobicity – Decreased particle size and Zeta potential (ζ‐potential)	Zhang, Liu, et al. (2024)

## 4 Cold Plasma and Plasma Activated Water (PAW)

### 4.1 Cold Plasma

#### 4.1.1 General Descriptions

The cold plasma treatment method is a novel nonthermal processing approach that uses mild operating temperatures without chemical reagents. Compared with conventional processing methods, it is less expensive and can maintain the nutritional value of food and enhance its functional properties (Kumar et al. 2022). In recent years, the cold plasma approach has emerged as an effective method in the food industry for eliminating hazardous and sporulating organisms from food products. It has been approved as a disinfectant for both foods and the surfaces that come into contact with them. This technique is known for its production diversity and prospective uses, including direct or indirect plasma, nonthermal effects, functional coatings, and plasma‐treated water, while maintaining food quality attributes without negative impacts (Sainz‐García and Alba‐Elías 2023).

#### 4.1.2 Effects of Cold Plasma Processing on Food Proteins

**FIGURE 3:**
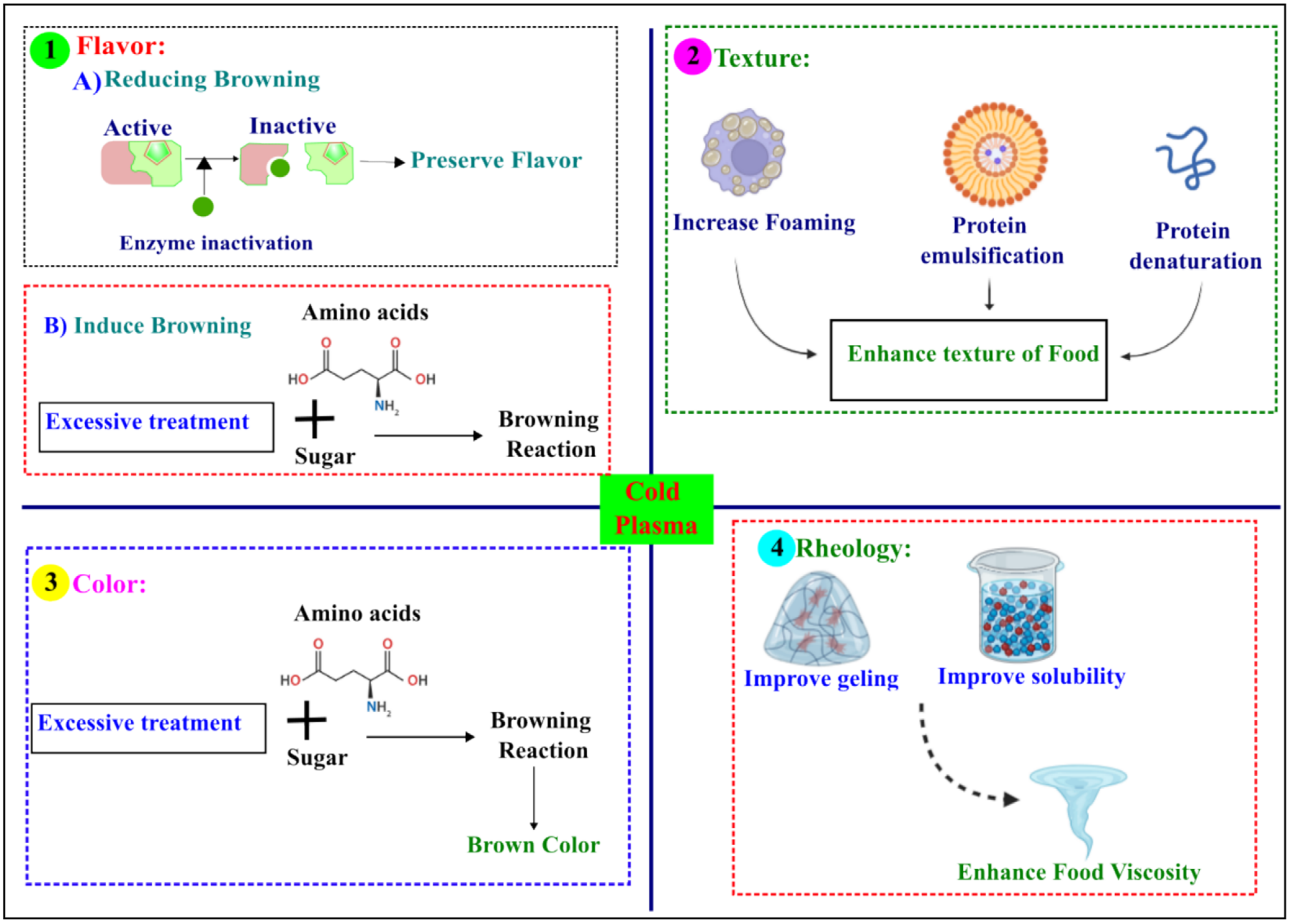
Major effects of cold plasma on food protein and their impact on food.

Cold plasma processing influences several components of food, such as lipids, proteins, carbohydrates, and bioactive substances. Proteins are a major component of food, and their structure, reactivity, foaming properties, emulsification properties, and solubility properties can affect flavor, texture, color, and rheology and are affected by cold plasma processing (Figure 3). Tan et al. (2024) reported that cold plasma increased the surface hydrophobicity of soybean protein isolates when increasing the treatment time. This could be due to the oxidative modification causing the protein structure to unfold, exposing hydrophobic groups buried at the interface of protein domains and subdomains or at the interface of subunits of oligomeric protein systems, thereby increasing the accessibility of sodium 8‐anilino‐1‐naphthalenesulfonic acid (ANS‐N). Cold plasma treatment increased the solubility of the protein by more than 30%, which could be due to the decrease in the particle size, leading to the exposure of a greater surface area to the solvent (Bu et al. 2022). According to their report, cold plasma also enhanced the functional properties (water and oil holding capacity, emulsifying activity and emulsion stability) of the material. When increasing cold plasma treatment time, an increase in the water and fat holding capacity was observed. However, a slight decrease in fat holding capacity was observed after treatment at 14 kV for 90 min. The emulsifying property, an important functional property of proteins that allows proteins to be used for texture improvement, was also improved following cold plasma treatment. Furthermore, the apparent viscosity of the protein isolate was highest at 14 kV after 90 min of treatment as a result of a decrease in droplet size and an increase in the surface area of the emulsion.

The cold plasma treatment led to a reduction in the particle size of sunflower seed protein, which was primarily attributable to the high energy imparted by the plasma impacting the protein surface, hence increasing the number of surface‐active sites. The smaller particles presented a greater number of active sites on the micellar surface, enhancing protein adsorption at the oil–water interface (Wang et al. 2024). The zeta potential, a measure of the stability of protein solutions, increased when increasing treatment time. These findings indicate that plasma treatment improved the stability of sunflower proteins because the reactive particles produced, such as N^2+^, O^3^, and H_2_O_2_, induced the depolymerization of soluble aggregates, exposing more polar groups on the surface of the protein and increasing the absolute value of the zeta potential. As reported, cold plasma treatment decreased the pH of milk, possibly because of the oxidation of polypeptides (long chains of amino acids) caused by ozone breakdown and the formation of acidogenic compounds (Sharma and Singh 2022). The prolonged treatment time resulted in the transformation of milk color to brown due to alterations in protein structure, potentially triggering the Maillard reaction, which is characterized by the covalent interaction between free ε‐amino groups in milk proteins and the carbonyl group of lactose (Chen, Wang, Guo, et al. 2022). Furthermore, the application of cold plasma led to alterations in the secondary structure, characterized by an increase in aggregated β‐sheet conformation alongside a concurrent reduction in the random coil structure of proteins, suggesting that aggregation occurs upon the unfolding of proteins as a consequence of cold plasma exposure.

The reactive oxygen species (ROS) and reactive nitrogen species (RNS) produced in cold plasma likely play significant roles in protein oxidation, altering the amino acid side chains and resulting in the disruption of noncovalent interactions, which ultimately affects the structural integrity of proteins (Luo et al. 2020). Some researchers have reported that ROS and RNS can react with various types of amino acids, highlighting the susceptibility of sulfur‐containing amino acids such as cysteine and methionine to ROS‐induced oxidation (Kehm et al. 2021; Wenske et al. 2021). It has been reported that cysteine can form disulfide bonds (‐S‐S‐) during oxidation, which can cross‐link protein chains and change the protein conformation. On the other hand, methionine can be oxidized to methionine sulfoxide, which strongly affects the hydrophobicity of the protein. In addition, reactive species may target various aromatic amino acids, including tryptophan, tyrosine, and phenylalanine. In particular, hydroxyl radicals (${\mathrm{OH}}^{.}$) can easily oxidize tryptophan, leading to the creation of oxidation products that alter the structure of the protein. The oxidation of amino acid side chains can significantly modify food protein structure (Andrés et al. 2022).

When food protein is subjected to cold plasma treatment, the oxidation of amino acid chains, which is reported to have a direct effect on food protein structure, unfolds in a sequential manner. Initially, oxidation induced by the reactive species generated by cold plasma targets and degrades α‐helices and β‐sheets, which are fundamental and crucial structural elements of the secondary structure of proteins. As the oxidation process progresses, it triggers the formation of disulfide bonds and other covalent cross‐links among protein chains. Simultaneously, it modifies noncovalent interactions such as hydrophobic contacts and hydrogen bonds. Bigger protein complexes are developed following aggregation, affecting the solubility, emulsifying, and foaming properties of food proteins (Heinonen et al. 2021).

Sharma and Singh (2022) examined the impact of cold plasma treatment on the rheological properties of milk and found cold plasma treatment improved gelation parameters such as the storage modulus (G′), gelation time, and gel texture, and reduced the average gelling time of skim milk from 21.0 to 12.8 min after 3 min of treatment. It is possible that some whey protein had already denatured, which could affect the gelling time of milk. Cold plasma treatment (1–2 min) reduced syneresis in skim milk. However, prolonged treatment resulted in syneresis due to the formation of larger casein micelle aggregates that clumped together, creating a poorly defined gel network and high levels of syneresis. Dharini et al. (2023) investigated the impact of cold plasma processing on sesame milk, and found that b* values steadily increased as the plasma power level increased, indicating increased browning caused by the Maillard reaction between protein and carbonyl groups. The lipoxygenase activity in raw milk, which is activated by the lipoxygenase enzyme, decreased from 100% to 67% after 120 W of plasma power treatment, demonstrating that the change in protein structure caused by cold plasma inhibited the activity of the lipoxygenase enzyme (Manoharan et al. 2023). Cold plasma treatment altered the order structure of protein and starch in raw sesame milk, resulting in changes in peak temperature and enthalpy, indicating thermal stability. Similar findings from cold plasma treatments of various foods have been reported (Held et al. 2019; Hu, Chen, et al. 2023; Markoska et al. 2019). Typical examples of the effects of cold plasma on food proteins are summarized in Table 3.

**TABLE 3:** Effects of cold plasma on food proteins.

Protein source	Cold plasma conditions	Major observations	References
Skim milk	– Voltage (20 kV) – Frequency (ranging from 15 to 25 Hz) – Treatment time (1, 2, 3, 4, and 5 min)	– ⍺‐lactalbumin and β‐lactoglobulin exhibited highest susceptibility to denaturation – Cold plasma treatment reduced the antibody binding capacity of protein	Sharma et al. (2022)
Phycocyanin	– Power (50 W) – Treatment time (0–180 s) – Temperature (30°C–60°C)	– Higher foaming capacities were recorded for samples treated for 60–80 s – Longer cold plasma treatment (40 and 60 s) resulted in a significant rise in average particle size (249.57 and 385.84 nm) – α‐helix content gradually decreased with increasing plasma treatment time	Gong et al. (2025)
Asian Sea Bass	– High voltage (230 V) – Frequency (50 Hz) – Treatment time (5, 10, and 15 min)	– Total Carbonyl Content (TCC) was increased from (1.16 to 7.93) nmol/g protein with increasing treatment time – Surface hydrophobicity increased at short treatment times but decreased as treatment time extended – Highest breaking force and deformation was recorded at 10 and 15 min treatments, respectively	Olatunde et al. (2021)
Shrimp	– Input voltage (50 V) – Input current (1 A) – Treatment time (0, 5, 10, 15, and 20 min)	– Free amino acid content increased by more than 70% – At 20 min treatment time, α‐helix decreased by 69% and the surface hydrophobicity increased by more than 57% – Immunoglobulin E (IgE) binding capacity decreased	Cheng et al. (2023)
Sheep milk	– Input power (40 W) – Processing time (30, 180, and 300 s)	– Remarkable change in protein secondary structure was observed – pH and color profile of the milk changed as a result of protein oxidation – β‐sheet and β‐turn structures increased – Protein aggregation was inhibited at 180 and 300 s processing time	Wang, Liu, et al. (2022)
Sunflower seed	– Power (50 W) – Current (1 ± 0.2 A) – Treatment time (0, 1, 2, 3, 4 and 5 min)	– Particle size was reduced – Zetapotential increased with increasing treatment time – Change in amino acid side chain and breakage of peptide bonds was observed – Solubility increased at 3 min, but decreased at 4 and 5 min treatment time	Wang et al. (2024)

### 4.2 Plasma Activated Water (PAW)

#### 4.2.1 General Descriptions

Plasma‐activated water (PAW) is water treated with cold plasma, and it contains reactive species such as nitric oxide radicals, hydroxyl radicals, superoxide anion radicals, atomic oxygen, singlet oxygen, nitrogen ions, and excited nitrogen molecules (Rahman et al. 2022). Like other nonthermal processing methods, PAW effectively inactivates various microorganisms (e.g., bacteria, fungi, and viruses), ensuring microbiological safety without significantly affecting the original quality or nutritional content of foods. Owing to its residue‐free process, low operating temperature, ease of preparation, and cost effectiveness, PAW has gained popularity for the decontamination of foods such as fruits, vegetables, and fish (Hosseini et al. 2020).

**FIGURE 4:**
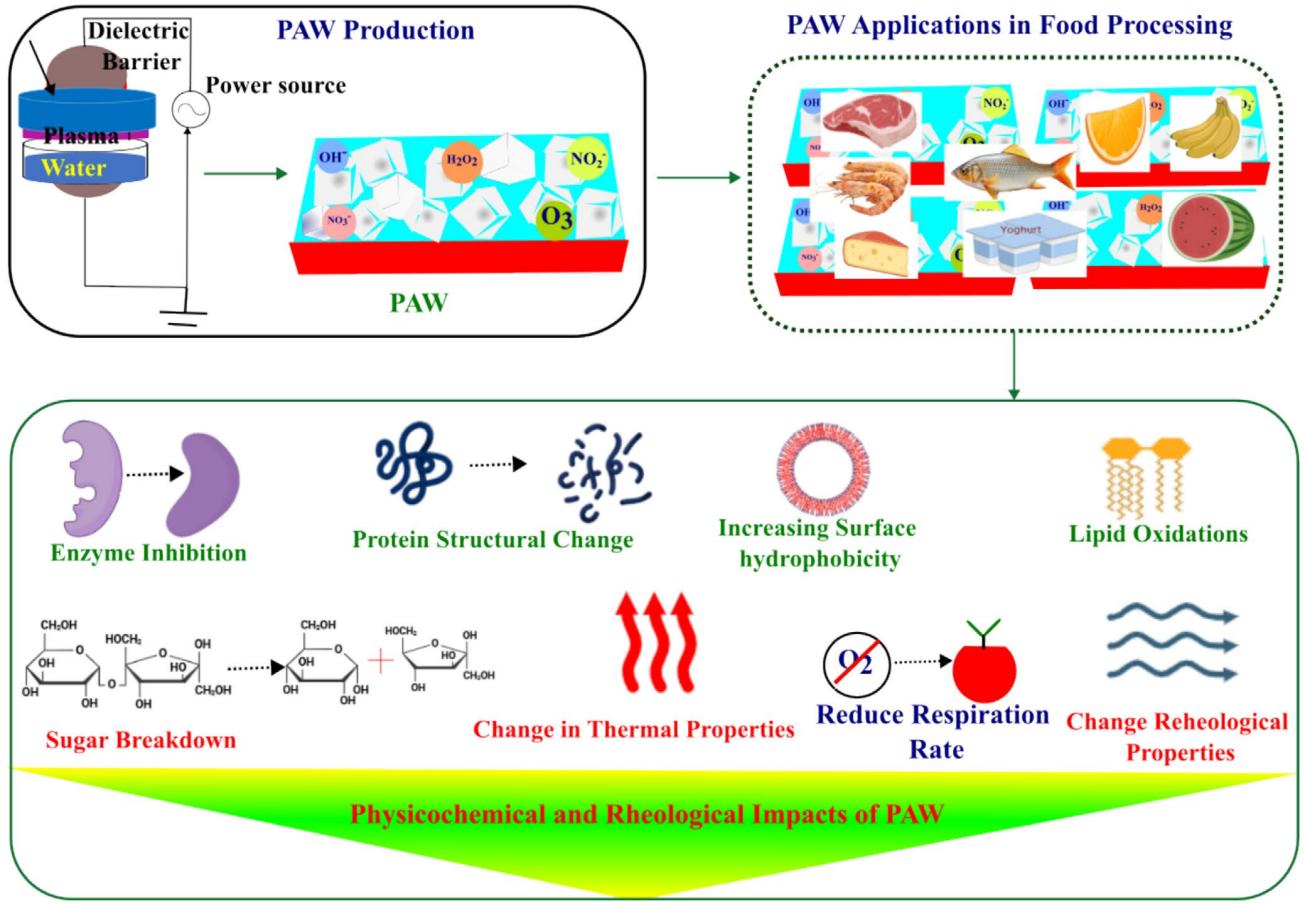
Effects of PAW on the physicochemical and rheological properties of foods.

Dielectric barrier discharge atmospheric cold plasma is frequently employed to produce plasma‐activated water (PAW) (He, Xie, et al. 2022). Water is activated by a plasma using input power from a high‐voltage power supply. The PAW is subsequently transferred to another storage and can be utilized for the preservation of meat, fish, shrimp, dairy goods, fruits, and vegetables, among other products (Figure 4). In addition to its antimicrobial properties, PAW has various impacts on physicochemical and rheological characteristics, including surface hydrophobicity, lipid oxidation, sugar degradation, alterations in thermal properties, a reduced respiration rate, and modifications in rheological properties. Thus, the properties of food protein change as a result of PAW treatment.

#### 4.2.2 Effects of Plasma‐Activated Water (PAW) on Food Proteins

A summary of recent studies on the impact of PAW on food protein is presented in Table 4. PAW treatment has the potential to alter the structure and functionality of many food proteins. Reports on whey protein isolates (Perinban et al. 2023), *Aristichthys nobilis* myofibrillar protein (Li, Shi, et al. 2022), bambara globulin (Alabi et al. 2022), and duck myofibrillar protein (Rao et al. 2023) have shown that PAW treatment induces mild oxidation, unfolding, and alterations in protein structure. The aforementioned modifications typically lead to changes in secondary structure, increased hydrophobicity, and improved functional qualities of proteins, including foam stability and gel strength (Rao et al. 2023). PAW treatment also increased protein aggregation and improved gelation characteristics (Jiang et al. 2023; Li, Rao, et al. 2024). While the effects vary depending on the protein source and treatment circumstances, (Alabi et al. 2022) reported that PAW treatment reduced emulsifying capabilities while increasing foaming properties.

**TABLE 4:** Effects of PAW processing on food proteins taken from different foods.

Source of protein	PAW conditions	Major findings	References
Corn flour	– Input power = 50 ± 1 W – Discharge frequency = 10 kHz – Optimal discharge time = 10 min – Combined with hydrothermal treatments	– Starch gelatinization and protein denaturation → starch‐protein aggregation → increasing particle size – Unfolding, dissociation, and rearrangement of the helical structures of the protein → disruption in the secondary structures of proteins, such as β‐turn and α‐helix	Zou et al. (2024)
Mozzarella cheese	– 75 kV for 2, 3, and 5 min, at room temperature	– A significant difference (*p* < 0.05) in protein content (Treated mozzarella showed relatively higher protein content) – Changes in protein matrix → improvements in taste and color of Mozzarella cheese	Nemati and Guimarães (2024)
Walnut kernels	– 19 kV, compressed atmospheric air at 22.5 L/min – Pulsed power supply at 20 kHz	– A significant difference in protein content among groups, but no significant difference in protein content with storage time (Showing PAW can preserve nutrition) – PAW retarded the increase in peroxidase (POD), polyphenol oxidase (PPO), and lactase (LA), while inhibiting the decrease in catalase (CAT) activity	Xiao et al. (2023)
Aristichthys nobilis (bighead carp)	– PAW was generated using high performance digitized plasma generator – Plasma jet exposition time: 0, 30, 60, 120, and 240 s – 300 mL of deionized water	– Protein solubility enhanced with PAW treatment for 30 and 60 s, but decreased for 120 and 240 s – PAW treatment reduced the proportion of α‐helix in myofibrillar proteins (MPs) from 31.61% to 26.95% (*p* < 0.05), indicating PAW altered the secondary structures of myofibrillar proteins, transforming them from α‐helix and random coil to β‐sheet and β‐turn – PAW treated for 30–120 s promoted the aggregation of myofibrillar proteins – PAW treatment for 120 s significantly (*p* < 0.05) increased the gel strength of myofibrillar proteins from 15.16 to 56.05 g mm – A drastic increase in storage modulus (G') was recorded after 237 s PAW treatment, indicating the improved gelling ability of myofibrillar proteins	Li, Shi, et al. (2022)
Bambara globulin	– PAW: pH 3.4, and conductivity of 200 mV – Treatment temperature: 4°C	– PAW treatment resulted in a slight redshift in fluorescence intensity, indicating protein structural unfolding, which was also connected with enhanced hydrophobicity – Following PAW treatment, emulsifying capacity of Bambara globulin was reduced, while foaming capacities were significantly better and stable	Alabi et al. (2022)

Li, Shi, et al. (2022) investigated the effect of PAW on myofibrillar protein and reported that PAW significantly weakened (*p* < 0.05) ionic connections while increasing hydrophobic interactions. This promoted protein aggregation and gelation, leading to the formation of a stronger gel and a denser three‐dimensional network. Furthermore, the results confirmed that increasing treatment time led to the transformation of α‐helixes and random coils to β‐sheets and β‐turns, as well as a constant increase in the storage module and a shorter protein degradation time. Compared with untreated protein, treatment of duck myofibrillar protein with PAW for 20 s enhanced both gel strength and water retention capacity (Rao et al. 2023). This improvement is attributed to the increased hydrophobic interactions between protein molecules, which resulted in a more uniform microstructure. Additionally, a higher level of PAW treatment led to increased concentrations of sulfhydryl and carbonyl groups. The enhancements in protein properties significantly impact the texture and sensory qualities of meat products (Chen et al. 2020; Xia et al. 2019). These improvements primarily address the limitations that hinder the development of duck meat products, such as lower emulsifying properties and weak gelling characteristics, which result in a rough taste and poor palatability of duck meat products (Wang, Li, Qu, et al. 2022).

The structural, physicochemical, functional, and digestive properties of corn flour modified with plasma‐activated water in combination with hydrothermal treatments have been studied (Zou et al. 2024). The study revealed that, for some of the samples, protein denaturation and starch gelatinization resulted in starch‐protein and starch‐starch aggregation, leading to an increase in particle size to some extent. In addition, PAW caused unfolding, dissociation, and rearrangement of the helical structure of the protein present in the corn flour. This process could enhance the interaction and breakdown efficiency of digestive enzymes on the protein, ultimately leading to improved protein digestibility and better absorption of amino acids (Zhu et al. 2021).

A study investigating the mechanisms and effects of PAW on myofibrillar protein from silver carp (*Aristichthys nobilis*) also reported that PAW accelerates protein aggregation. Treatment with PAW for 60 s using a plasma spray gun resulted in the densest and most homogeneous cross‐linking morphology. Compared with those of the untreated samples, the intensities of the myosin heavy chain (MHC), actin, and tropomyosin bands were significantly lower in the samples treated with PAW. The proportion of α‐helices decreased markedly (*p* < 0.05), dropping from 52.14% in the PAW_0_ sample to 28.22% in PAW_240_. Meanwhile, the levels of β‐sheet and β‐turn increased, with β‐sheets rising from 18.68% to 36.25% and β‐turn from 6.39% to 16.79% (*p* < 0.05). These findings indicate that PAW promotes the transformation of secondary structures of muscle proteins from α‐helices to β‐sheets and β‐turns. While most amino acids in PAW‐treated samples slightly decreased, it can be concluded that PAW is effective for modifying food proteins to enhance their functional properties, particularly their gelling abilities, thus improving protein‐based products (Xiong et al. 2021).

## 5 High‐Pressure Processing (HPP)

### 5.1 General Descriptions

Among the unique and alternative methods, high‐pressure processing (HPP) technology has become popular as a preservation method in the food processing industry. High‐pressure processing is a promising and safe technology for protein structure modification. Pressure processing can cause protein denaturation, aggregation, or gelation on the basis of the protein system, treatment temperature, and solution conditions (e.g., pH and ionic strength) (Sahil et al. 2024). HPP is effective at preserving foods, including liquids, semisolids, and solids. It is well known for its powerful antibacterial impact while maintaining the nutritional quality of food (Houška et al. 2022). The efficacy of HPP is influenced by various factors, including pressure magnitude, holding period, temperature, compression rate, and the intrinsic qualities of the food. Ineffectiveness for some dairy, animal, and shelf‐stable low‐acid goods is regarded as its principal drawback (Woldemariam and Emire 2019). Moreover, a number of variables may affect the procedure, with different foods needing varying amounts of pressure to be preserved (Nema et al. 2022). In this section, the effects of high‐pressure processing on the rheological, functional, and structural properties of proteins are summarized.

### 5.2 Effects of HPP on Food Proteins

Ribeiro et al. (2018) studied the physical properties of fish ham subjected to HPP and found that the whiteness of the fish ham increased with increasing the pressure, which was mostly related to the degree of protein denaturation, particularly myoglobin denaturation. A pressure of 350–500 MPa at 30°C resulted in significant changes in the textural qualities of the ham, indicating improved protein gelling properties. Rajan et al. (2023) reported that HPP reduced α‐helix fractions in whole soymilk from 29.6% (control) to a minimum of 10.3% (600 MPa, 5 min) by disrupting the hydrogen bonding between the C=O and N—H bonds of protein peptide chains. After α‐helix denaturation, the fraction of β‐sheets increased from 32.0% (control) to 48.1% (600 MPa, 5 min), indicating that HPP can alter the secondary structure of proteins by altering amino acid side chains and forming new bonds through unfolding. Furthermore, HPP increased the viscosity of soymilk from 4.13 to 6.22 mPa.s at 600 MPa for 15 min, indicating that HPP induced aggregation and protein structural changes. As HPP intensifies, soymilk viscosity steadily increases due to increased protein water holding capacity.

HPP improves the oil absorption and emulsification of red quinoa gel by dissociating and unfolding polypeptides, exposing hydrophobic amino acid sites (Thakur et al. 2024), which allows for the hydrophobic attachment of peptide chains to lipid droplets, increasing the volume and surface area of proteins available for interaction and improving oil absorption and emulsification. The gel strength of the quinoa gel slightly increased, possibly due to protein denaturation, resulting in increased aggregation. A maximum increase in the particle diameter of the gel at 600 MPa was also reported by (Muñoz et al. 2023), which could be due to a disruption in the micellar structure. The protein aggregation increased with pressure, peaking at 450 MPa and reaching high levels at 600 MPa. Cropotova et al. (2020) reported that HPP treatment at 200 and 300 MPa reduced the solubility of sarcoplasmic proteins in haddock mince by 0.4% and 42.0%, respectively, compared with that of control samples. In addition, myofibrillar protein solubility decreased in both haddock and mackerel minces. Compared with the haddock mince treated at 200 MPa, the haddock mince treated at 300 MPa had a greater loss of protein solubility.

Pressurizing haddock and mackerel minces at 200–300 MPa for 5 min reduces the functioning of sarcoplasmic and myofibrillar proteins, resulting in lower solubility due to the formation of insoluble aggregates (Chen, Wang, Zhu, et al. 2022). High pressure causes weak noncovalent bonds to rupture, resulting in protein denaturation and aggregation and the development of new intra‐ and/or intermolecular noncovalent links (Castrica et al. 2021). After 1 month of frozen storage, the carbonyl content (a crucial indicator of protein oxidation) increased significantly in both the sarcoplasmic and myofibrillar proteins of pressurized fish minces compared with those of untreated minces. The undesirable impact of HPP on protein oxidation in fish minces during frozen storage can be attributed to increased membrane damage compared with that in controls (Puértolas and Lavilla 2020). Tsevdou et al. (2023) also reported that high‐pressure treatment ruptures adipocyte cell membranes, releasing lipid oxidation products that interact with enzymes and cause protein oxidation. HP treatment resulted in higher b* values in haddock and mackerel minces than in the control samples, which could be attributed to the accumulation of secondary lipid oxidation products, such as those formed by the interaction of aldehyde groups with free amino groups in phospholipids and proteins (Tatiyaborworntham et al. 2021). Compared with that of the control samples, the L* values of both the HP‐treated haddock and mackerel samples increased with increasing hydrostatic pressure, indicating that the color of the fish decreased as the hydrostatic pressure increased due to conformational changes in the proteins. Furthermore, fishcakes made from HP‐treated haddock and mackerel minces had lower hardness and cohesiveness than untreated minces did. This could be due to pressure‐induced degradation of structural proteins such as myosin and actin, which may be aided by proteolytic enzymes released from HP‐damaged cells. Moderate HPP (< 400 MPa) can activate proteolytic enzymes such as cathepsins B and D in fish tissue, leading to protein degradation. The tenderness of fish tissue during HP‐pressure treatment is a result of both high pressure and proteolytic enzyme activity (Arnaud et al. 2018). Compared with the control, the HPP treatment resulted in lighter and more crimson crab flesh gels. At 600 MPa, HPP caused myosin heavy chain denaturation and aggregation, resulting in large molecular aggregates. Compared with those at other pressure levels, the α‐helix structures shifted to β‐sheets and β‐turns at 100 MPa. Medium‐pressure treatment increased the gelling capacity of crab meat and the texture by changing the protein structure (Martínez‐Maldonado et al. 2020).

Mbye et al. (2021) investigated the combined effects of pasteurization and HPP on the quality of camel and bovine cheese. The antiviral and antibacterial properties of camel milk are attributed to the presence of peptidoglycan recognition protein (PGRP) enzymes, immunoglobulins (Igs), N‐acetyl‐β‐glucosaminidase (NAGase), lactoferrin (LF), lactoperoxidase (LP), and lysozyme (LZ) (Karaman et al. 2021). The authors claimed that high‐pressure processing (HPP) enhances cheese yield by promoting the denaturation of whey proteins, particularly β‐lactoglobulin. This denaturation facilitates interactions with casein micelles, leading to the formation of barriers that inhibit the reformation of casein aggregates during curd formation. As a result, the cheeses present an open structure and increased moisture content, ultimately contributing to a higher yield. The authors also confirmed that increased water retention resulting from the hydration of the protein network led to a reduction in cheese firmness. Water within the protein matrix functions as a plasticizer, reducing elasticity and increasing susceptibility to fracture under compression. Ahmad et al. (2024) reported that noncovalent bonds, which are influenced by high‐pressure processing (HPP) in terms of compressibility, appeared to be the primary cause of alterations in the UV spectra of oat milk protein extracts, as opposed to covalent bonds. Oat proteins contain hydrophobic and aromatic amino acid residues, including tryptophan and tyrosine. A summary of some examples of the effects of HPP on food proteins is presented in Table 5.

**TABLE 5:** Some examples of the effects of HPP on food proteins.

Protein sources	HPP process variables	Major findings	References
Fish ham	– Pressure levels (200, 350, and 500 MPa) – Pressurization times (10 and 20 min) – Temperatures (10°C and 30°C)	– Protein (mainly myoglobin) denaturation resulted in increase in whiteness – HPP treatment at 500 MPa/10 min/30°C decreased water‐holding capacity – Hardness, gumminess, cohesiveness, and chewiness of the protein gel was improved	Sakamut et al. (2025)
Sheep milk	– Applied pressure (150, 300, 450, and 600 MPa) – Constant time and temperature (5 min and 10°C, respectively)	– Maximum increase in mean particle diameter was measured at 600 MPa – Protein dispersion instability reached higher at 450 MPa – Medium pressure (300 MPa) treatment improved coagulation kinetics	Muñoz et al. (2023)
Crab meat	– Applied pressure (100, 300, and 500 MPa) – Constant time and temperature (5 min and 10°C, respectively)	– The thermal transition showed sample treated at 500 – MPa did not present an endothermic peak (*T*_max_), suggesting that myosin was completely denatured – α‐helices decreased gradually from 43.48% (untreated) to 24.57% at (500 MPa) – β‐sheet increased from 30.11% (untreated) to 45.70% (500 MPa) – Proportion of hydrogen bonds were higher on pressurized samples, indicating changes in the protein conformation and gel structure	Velazquez et al. (2021)
Barley‐based milk alternative	– Applied pressure (100, 300, and 600 MPa) – Number of pulses (1, 2, and 3 at 100 and 300 MPa) – Temperature (40°C and 80°C)	– Higher pressure pulses at 100 MPa resulted homogenous system – A 300 MPa pressure promoted higher protein solubility than other samples	Strieder et al. (2022)
Bovine milk	– Pressure levels (200–600 MPa) – Temperature (20°C)	– After 10 min of treatment, protein coagulation was observed for all untreated and treated samples – Samples treated at 200 MPa had almost identical protein profiles – HPP (600 MPa) treatment resulted in more rapid hydrolysis of the caseins	He, Yang, et al. (2022)
Red quinoa	– Pressure levels (200, 300, 400 and 500 MPa) – Germination (48 h)	– HPP and germination resulted protein degradation → increased the swelling capacity of flour – Partial loss of protein structure at high pressure resulted in a decrease in residual enthalpy	Thakur et al. (2024)

## 6 Pulsed Electric Field (PEF)

### 6.1 Working Principles

**FIGURE 5:**
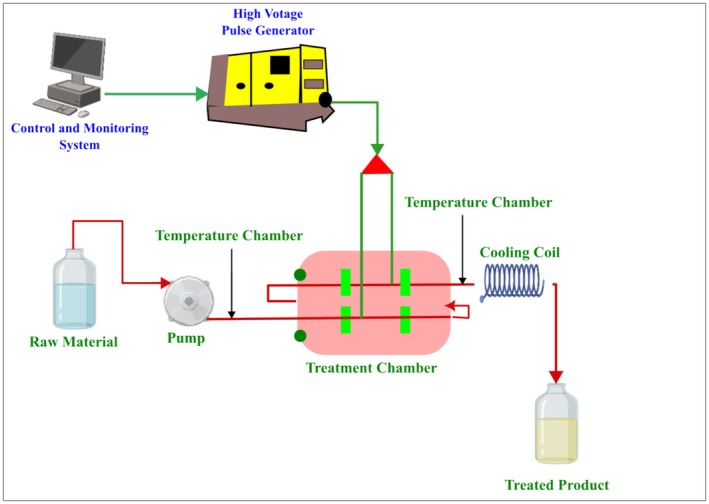
Basic components of pulsed electric fields in food processing (Raso et al. 2022).

A simple pulsed electric field processing system consisted of a high‐voltage power supply, an energy storage capacitor bank, a charging current limiting resistor, a switch to discharge energy from the capacitor across the food, and a treatment chamber (Figure 5). The pulse waveform is monitored via an oscilloscope. A high‐voltage DC generator converts voltage from a utility line (110 V) into a high‐voltage alternating current (AC), which is then rectified to a high‐voltage direct current (DC). Energy from the power supply is stored in the capacitor and discharged through the treatment chamber, creating an electric field in the food product. The voltage across the capacitor is intended to be equivalent to the voltage across the generator. To charge the capacitor bank, a direct current power source is utilized, which is generated by amplifying and rectifying the regular alternating current. Energy is discharged from the capacitor storage bank across the food in the treatment chamber via an electrical switch (Raso et al. 2022).

PEF is quickly gaining popularity as a promising food processing technology because of its low‐temperature requirements, short processing time, and environmental friendliness. In contrast to conventional food processing methods, PEF minimally impacts food nutrition, effectively eradicates harmful microbes, and extends the shelf life of food products (Rahaman et al. 2020). Several studies have reported the use of PEF in food processing, including active substance extraction, pasteurization, drying, and thawing of food products, and modifications to the physicochemical, rheological, and functional properties of foods (Dong et al. 2020; Hu et al. 2024; Walkling‐Ribeiro et al. 2024; Wang et al. 2023). PEF has the potential to induce structural changes, increase free amino acids, improve functional properties, and increase the recovery of proteins in foods (Taha et al. 2022).

### 6.2 Effects of Pulsed Electric Fields (PEFs) on Food Proteins

A summary of the effects of PEF on proteins derived from different foods is presented in Table 6. The sources of food protein, electric field profile, major findings, and corresponding references are provided. PEF treatment increased the solubility of micellar casein from 84.9% (untreated) to 87.1% (treated at 10 kV/cm). The molecular polarization and better dielectric constant that occurred from the PEF treatment are the main causes of the increased solubility of micellar casein (Taha et al. 2023). PEF treatment reduced the α‐helix content and increased the β‐sheet content, indicating changes in secondary protein structure and hydrogen bonding. Compared with ultrahigh‐temperature (UHT) and low‐temperature long‐term (LTLT) processing, PEF treatment has a weaker effect on the content of β‐lactoglobulin in milk (Šalaševičius et al. 2021). Furthermore, after PEF treatment, the undenatured whey protein concentration remained unchanged compared with that of the raw milk samples. Manzoor et al. (2019) reported a decrease in the apparent viscosity of almond milk, which was mostly due to PEF processing, causing alterations in almond milk protein coagulation, fat globule size, and distribution. Increasing the PEF intensity from 7 to 28 kVcm^−1^ resulted in considerably higher apparent viscosity (*p* < 0.05), indicating intermolecular interactions between neighboring denatured molecules due to weak transient networks. PEF treatment at 2.8 kV resulted in an increase in the denaturation temperature (T_peak_), indicating improved polypeptide interaction and the ability to form a rigid self‐supporting gel without complete protein denaturation (Table 6).

**TABLE 6:** Effects of PEF on proteins from different food sources.

Source of protein	PEF parameters	Major findings	References
Egg white	– Electric field profile (pulse width: 3–15 μs; frequency: 50–1000 Hz; and voltage: 0–35 kV). – Residence time (1, 2, 3, 4, and 5 min)	– No significant change in molecular size of egg white protein – Voltage increases the UV absorption intensity from 0.569 of the control to 1.021 – ↑ Voltage and residence time → *α*‐helices ↓, *β*‐sheets ↑ – Sulfhydryl groups increased (change in tertiary structure)	Qian et al. (2019)
Canola seeds	– Electric field profile (voltage: 10–35 kV at 5 kV interval; pulse frequency: 600 Hz; pulse width: 8 μs). – Residence time (60–210 s at intervals of 30 s)	– PFE significantly (*p* < 0.01) increased solubility of Canola seed protein from 43.25%–50.02% – Lower PEF parameters improved water‐holding capacity of Canola seed protein – Oil holding capacity increased from 8.12–13.19 mL/g – The emulsifying capacity and emulsion stability ↑ at 30 kV and 180 s – Foaming capacity ↑ (168%) and foaming stability ↑ (92%) – Resulted more organized secondary structure of canola protein – Free Sulfhydryl groups ↑ and total sulfhydryl groups ↓	Zhu et al. (2024)
Milk whey	– Electric field intensities (EFIs, 5–20 kV/cm) – Pulse frequency: 50 Hz; pulse width: 10 μs – Treatment times (2–8 ms)	– Solubility > 10%↑ at 20 kV/cm – No major variation in the protein composition. – < 10 kV/cm treatment) unfolds protein structure – 20 kV/cm for 8 ms → particle size ↓, zeta potential ↑, carbonyl concentration ↑ – Prolonged treatment improved emulsifying properties	Hu et al. (2024)
Beef	– Electric field strength (1.00–1.25 kV/cm) – Constant pulse width of 20 μs – Frequency: 50 Hz	– PEF improved protein digestibility by at least 18% – Protein profiles of the muscle were not modified – No significant difference in thermal profile of the protein	Chian et al. (2019)
Soy	– PEF strength: 5, 10, and 20 kV/cm – Pulse frequency: 1000 Hz – Pulse width: 40 μs	– Highest water solubility (36.7%) at 10 kV/cm – No significant different in amino acid compositions – The zeta potential increased up to 50.2 mV – A slight increase in foaming capacity (18.7%) was observed – Higher treatment caused. aggregation and oxidations	Wang et al. (2023)
Beef	– PEF profile: 5 kV, 90 Hz, 20 μs (T_1_); 10 kV, 20 Hz, 20 μs (T_2_) – Subjected to in vitro gastrointestinal digestion	– The protein digestibility (%) at (10 kV, 20 Hz) – Relatively higher in amino acid values were observed following PEF treatment – Soluble protein (%) of the treated samples were significantly (*p* < 0.05) higher than the control	Bhat et al. (2018)
Casein micelles	– Applied voltage: 2, 4, and 6 kV (10, 20, and 30 kV/cm, respectively) for 10 pulses – Current values: 360, 680, and 1060 A (for 10, 20, and, 30 kV/cm, respectively)	– 20–30 kV/cm electric field → ↓ particle size of proteins. – Absolute ζ‐potential value increased – Maximum protein solubility (87.1%) observed at 10 kV/cm of PEF treatment – It caused alterations in the conformational structure of the protein	Taha et al. (2023)

Cropotova et al. (2021) studied the impact of pulsed electric field pretreatment on sea bass quality parameters and reported that a lower pH was obtained compared with that of untreated samples, which could be due to ion release from disrupted cells or structural changes in proteins that allow for acidic group release. No significant differences in water holding capacity and water activity between PEF pretreated and untreated samples were reported. More studies have investigated the effects of PEF treatment on food proteins (Dong et al. 2020; Mungure et al. 2020; Šalaševičius et al. 2021; Shiekh et al. 2020; Taha et al. 2023).

**FIGURE 6:**
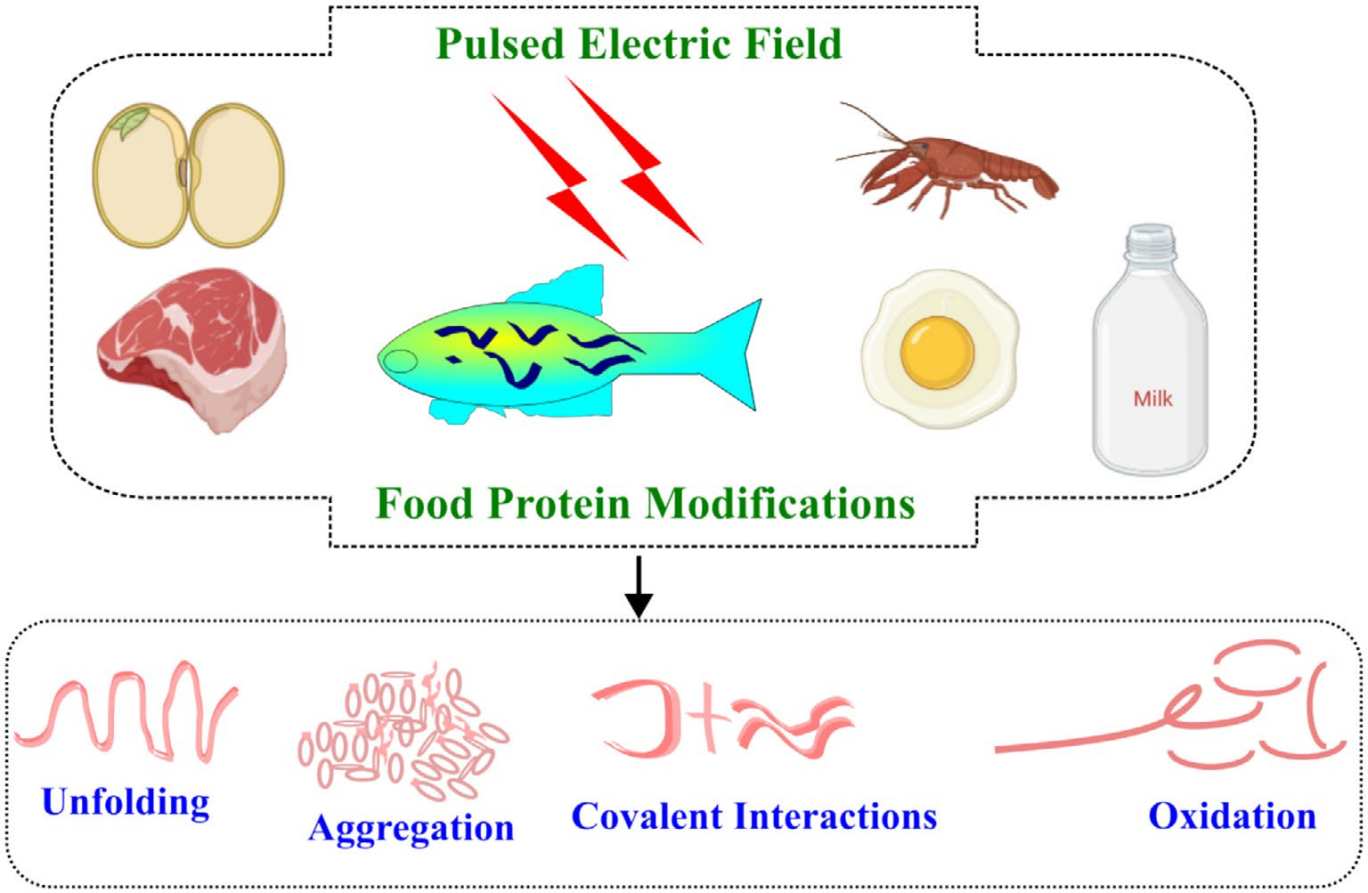
Mechanisms of food protein modifications induced by PEF (Shams et al. 2024; Taha et al. 2022).

A general representation of the mechanisms of food protein subjected to PEF treatment, highlighted by (Shams et al. 2024; Taha et al. 2022), is depicted in Figure 6. The mechanism of food protein modification under PEF treatment includes, but is not limited to, unfolding, aggregation, covalent interactions, and oxidation. Initially, PEF disrupts the tertiary and quaternary structures of proteins via electrostatic forces, leading to protein unfolding and the exposure of hydrophobic areas, hence increasing their susceptibility to subsequent interactions. Following unfolding, the exposed hydrophobic areas of the proteins interact with each other, causing aggregation and the development of protein aggregates that can alter the texture and consistency of the food, which is useful for texture modification in the final food products (Wang, Li, Guo, et al. 2022). Furthermore, PEF could promote the development of covalent bonds between protein molecules, such as disulfide bonds, thereby stabilizing aggregation and modifying the functional properties of proteins, such as their solubility, gelling, and emulsifying capacity, all of which are important in food processing. Moreover, the electric field generated by PEF can promote oxidation reactions by creating free radicals that target protein side chains, resulting in oxidation. This oxidation can affect protein stability and functionality, typically resulting in changes in the flavor, color, and nutritional qualities of the food (Taha et al. 2022).

## 7 Enzymatic Hydrolysis

**FIGURE 7:**
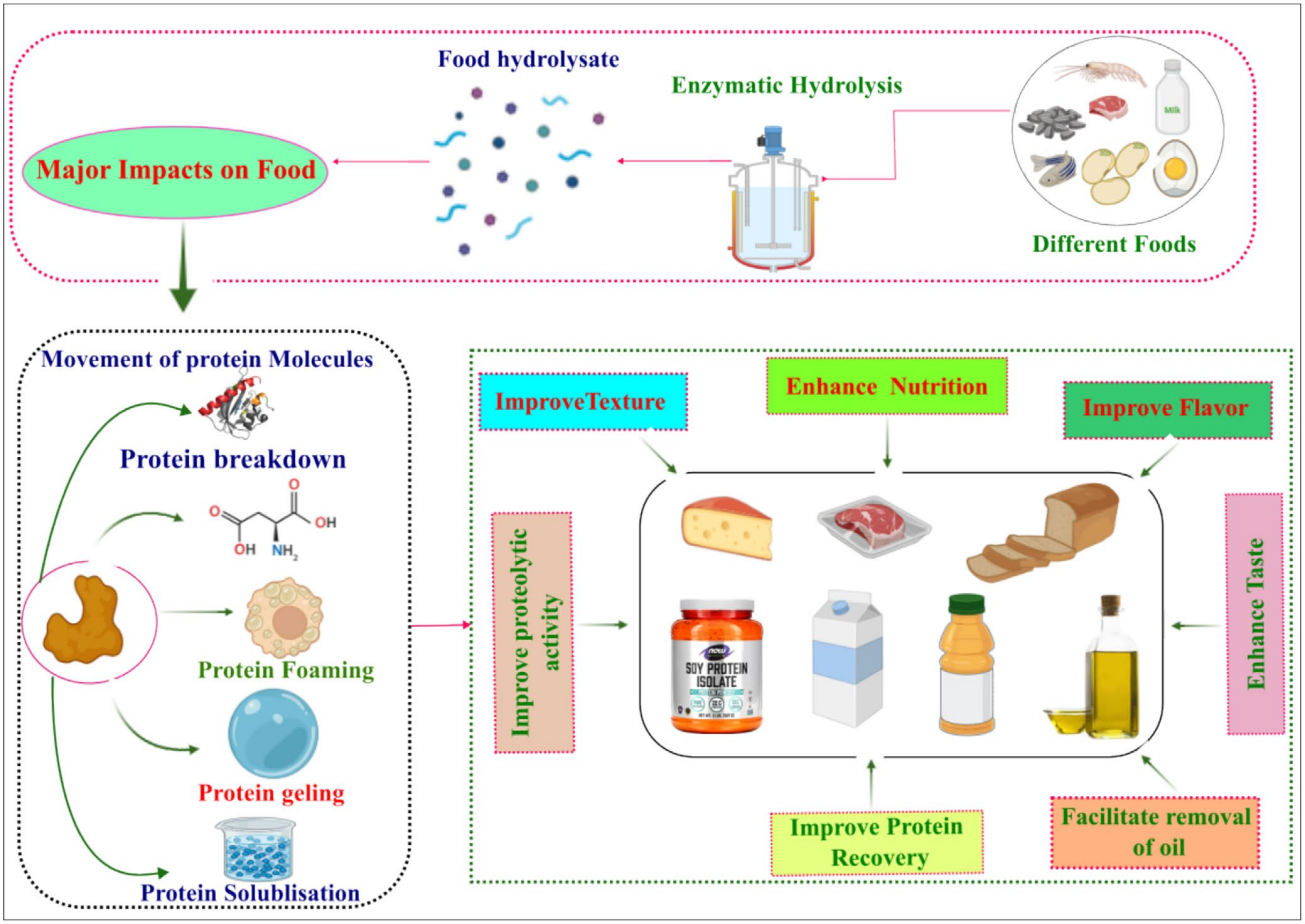
Impact of enzymatic hydrolysis on food proteins and final products.

Enzymatic hydrolysis is a novel food processing technique that breaks down food macromolecules via the use of enzymes and water. This process has the potential to alter the molecular structure of food constituents such as proteins, carbohydrates, and lipids (Boukid et al. 2023). Enzymatic hydrolysis has numerous effects on food protein, including improved texture, proteolytic activity, nutrition, flavor (Vogelsang‐O'Dwyer et al. 2022), and peptide recovery (Chen et al. 2024), as well as the removal of oil from oil seeds and other oil sources (Zhao et al. 2023) (Figure 7).

Significant advances have been revealed in the enzymatic hydrolysis of food proteins and their sources, including snacks, meats and meat substitutes, sauces and soups, bakery items, desserts and ice cream, and beverages (Mohammadi et al. 2022; Zhao et al. 2021). Various factors influence the enzymatic hydrolysis of foods. Substrate factors such as physical form, particle size, and fiber content strongly impact hydrolysis rates (Tang et al. 2023). Enzyme diffusion, substrate porosity, enzyme adsorption, and catalytic events all contribute to hydrolysis limitations. pH, ionic strength, heat, and mechanical shear influence protein hydrolysis.

A summary of the effects of enzymatic hydrolysis on various food proteins is presented in Table 7. Gu et al. (2021) processed egg yolk powder via enzymatic hydrolysis and supercritical fluid extraction. An alcalase enzyme concentration of 500 U/g, an extraction temperature of 313.15 K, a period of 120 min, and a solid–liquid ratio of 1:9 (g/mL) were utilized. The degree of protein hydrolysis increased with increasing enzyme concentration, reaching a maximum (11.2%) at 2000 U/g. Compared with the untreated samples, the enzymatic hydrolysis‐treated samples presented more regular distributions of particle size, indicating a more uniform size. Enzymatic hydrolysis reportedly enhances protein solubility in egg yolk powder by converting insoluble proteins, such as high‐density lipoprotein (HDL), into peptides with lower molecular weights and greater hydrophilicity. Breaking peptide bonds exposes more polar groups and increases the number of hydrogen bonds between peptides and water molecules, leading to increased protein water solubility (WS). Hydrolysis modifies the triple helix configuration of gelatin and reduces the protein's molecular weight, hence affecting its functional characteristics. Hydrolysis exposes active groups such as carboxyl and amino‐terminal groups. Additionally, peptides become more hydrophilic and interact with water via hydrogen bonds (Sow et al. 2019).

**TABLE 7:** Effects of enzymatic hydrolysis on some food proteins derived from various foods.

Protein source	Enzymatic hydrolysis parameters	Major observations	References
Shrimp head	– Enzyme (Papain) – Enzyme concentration treatments (control, 1%, 2%, and 3%)	– Addition of 3% of papain enzyme resulted highest degree of protein hydrolysis – Soluble protein content increased with increasing papain enzyme concentration	Dzikri Hisyam et al. (2024)
Chlorella	– Enzymes (pepsin, alkaline, trypsin, and pancreatic enzymes) – Enzyme concentrations (2%)	– Trypsin and pepsin hydrolysates showed the highest and lowest degree of hydrolysis values, respectively – All hydrolysates exhibit more than 50% protein solubility – Highest water holding capacity (5.7) was observed for proteases hydrolysates	Gharehbeglou et al. (2024)
Soybean	– Enzyme (protease) – Temperature and pH (55°C and 9.0, respectively)	– Enzymatic hydrolysis reduced the particle size – The total amino acid increased from 37.4 to 39.2, 39.5, and then decreased to 38.6 mg/g with prolonged hydrolysis – Foaming properties increased as the degree of hydrolysis increased	Zhang, Du, et al. (2023)
Chicken viscera and bones	– Enzymes (alkaline endoprotease and microbial endoprotease) – Enzyme concentrations (0.1% w/w)	– Despite low enzyme activity, protein solubility was increased at high temperature (55°C) – Granulation and gel formation has been observed above 50°C	Five et al. (2024)
Egg yolk	– Enzyme (neutral and alkaline protease) – Enzyme concentration (2000 U/g egg yolk protein) – Reaction temperature and time (50°C and 4 h, respectively)	– Surface hydrophobicity of hydrolyzed egg yolk protein ↓, protein solubility ↑ – Hydrolyzed egg yolk exhibited smaller particle diameter than the original → better emulsifying stability – Hydrolyzed egg yolk protein had higher thermal stability	Gao et al. (2019)
Starry triggerfish	– Enzyme (trypsin) – Enzyme level (5.5% w/w) – Reaction conditions (pH 8.5 and 55°C) for 40 min	– Enzymatic hydrolysis increased solubility of hydrolysates to more than 70% – Emulsifying activities ↑ with enzyme conc. ≤ 2% but ↓ with conc. ≥ 3%	Sripokar et al. (2019)

Gao et al. (2019) reported that enzymatic hydrolysis of egg yolk resulted in an increase in gelling points from 78°C (untreated) to 90°C (enzymatically hydrolyzed), with a sharp increase in the storage module (G′) and loss module (G″), indicating an improvement in thermal stability. At each protein concentration, the emulsion prepared from hydrolyzed egg yolk had a smaller particle diameter than the original emulsion did, suggesting that the enzymatically hydrolyzed egg yolk had better emulsifying stability. Enzymatic hydrolysis modified the quantity and diversity of amino acids present in the yolk protein, hence affecting the protein charge density.

A two‐stage hydrolysis process was developed for industrial application at 55°C, consisting of 12 h of hydrolysis with neutral protease followed by 12 h of hydrolysis with aminopeptidase (Zhao et al. 2021). This method effectively generates protein hydrolysates that are rich in umami‐tasting and umami‐enhancing peptides, such as Asn‐Pro and Ala‐His, from nonsoy sauce protein preparations, including soy protein isolate, rice proteins, wheat proteins, peanut proteins, and pea proteins An increase in the thermal properties of oat core flour was noted after enzymatic hydrolysis; specifically, oat protein exhibited significant thermostability, with a denaturation temperature of approximately 110°C (Zhang, Huang, et al. 2024). The thermal properties of a protein are important in determining its resistance to aggregation/denaturation during the thermal process. At pH values below 7.0, the zeta potentials of both oat core flour and dual‐enzyme‐treated samples decreased dramatically, which was due to the precipitation of oat protein. The enzymatic hydrolysis of chicken carcasses removes fat and insoluble materials, resulting in a 21.38% increase in crude protein content/recovery (Zhang, Li, et al. 2023). Following enzymatic hydrolysis, the essential amino acids in chicken carcasses increased dramatically. These results suggest that higher levels of free amino acids and oligopeptides improve taste, including brothy, meaty, and savory flavors associated with preservation and hydrolysis (Begum et al. 2020).

Protein hydrolysates from starry triggerfish muscle were found to have greater DPPH radical scavenging activity at concentrations of 30–40 mg/mL (Sripokar et al. 2019). Enzymatic hydrolysis significantly increased protein solubility, reaching a maximum of 94% at pH 3. This change in solubility can be attributed to the net charge of the amino acid residues after hydrolysis, which increases as the pH moves away from the isoelectric point, promoting the aggregation of hydrophobic interactions (Dinakarkumar et al. 2022). In colloidal systems, soluble proteins give molecules a uniform dispersibility and improve the interfacial characteristics. Enzymatic hydrolysis can influence the size, hydrophobicity, polarity, and ionizability of protein hydrolysates. Another major factor influencing protein solubility is the balance of hydrophilic and hydrophobic peptide heads (Sinthusamran et al. 2020). Following enzymatic hydrolysis treatment, protein solubility increases exponentially, potentially affecting the appearance, sedimentation, viscosity, and flavor of fish during storage as well as mouth feel, digestibility, and metabolic responses during consumption (Cosson et al. 2022).

Enzymatic hydrolysis reduced the particle size of the soybean meal hydrolysates, indicating that the peptides were produced as a result of peptide bond breaking (Zhang, Du, et al. 2023). The total amino acid content increased from 37.4 to 39.2 and then to 39.5 before decreasing to 38.6 mg/g as the degree of hydrolysis (DH) increased. During the initial hour of hydrolysis, the amino acid concentration increases, indicating the degradation of protein into more amino acids; however, with prolonged hydrolysis, amino acid residues become re‐encapsulated inside the protein structure (Li, Yang, et al. 2022). SEM analysis revealed protein degradation caused by enzymatic hydrolysis. The shift in the amide I band from 1660 to 1650 cm^−1^ indicates a considerable difference in secondary structure, with fewer α‐helices and more β‐turns. Enzymatic disintegration improved the emulsification ability in a short period of time (1 h). The exposure of hydrophobic groups may reveal the interior mask of the molecule, enhancing the hydrophilic balance and emulsification ability. Sensory panelists rated soybean meal hydrolysates as bitter compared with soybean meal. The antioxidant activities of soybean meal subjected to enzymatic hydrolysis significantly increased (*p* < 0.05), likely due to the generation of small‐molecule peptides and the increased availability of active amino acids during the hydrolysis process. Increased levels of amino acids, including Thr, Asp, Lys, Glu, Met, and Gly, can effectively eliminate free radicals. More studies on the effects of enzymatic hydrolysis on food proteins have been reported (Bui et al. 2021; Gautam et al. 2024; Rios‐Morales et al. 2023).

## 8 Analyzing Variability in Results and Factors Affecting Food Protein Modifications

Different reports covered in this review revealed notable differences in various properties of food proteins subjected to novel food processing under similar conditions. For example, HPP increased protein solubility in barley‐based milk alternatives at 300 MPa (Strieder et al. 2022), but a (200–300 MPa) pressure decreased the solubility of protein from Haddock mince. The difference in solubility changes can be attributed to the distinct protein compositions and interactions in these two food sources. Haddock mince has a mix of muscle proteins, and the use of high pressure might lead to the creation of clumps with low solubility, especially at relatively higher pressures (Chen, Wang, Zhu, et al. 2022). In barley‐based milk alternative, the proteins might have a more flexible structure that allows for better dispersion and increased solubility under high pressure (Navare et al. 2023). HPP treatment at 500 MPa/10 min/30°C decreased the water holding capacity and improved the hardness, gumminess, cohesiveness, and chewiness of protein in fish ham (Sakamut et al. 2025). In crab meat protein, medium‐pressure treatment (300 MPa) increased the gelling capacity and texture. However, at 500 MPa, myosin was completely denatured, and the thermal transition showed no endothermic peak (Velazquez et al. 2021). The observed variations are likely attributable to differences in the types of proteins and their initial states in fish ham and crab meat. Fish ham may possess a more heterogeneous protein matrix, and high pressure can induce a more complex rearrangement of proteins, influencing water holding capacity and texture (Cropotova et al. 2020). Crab meat proteins, on the other hand, exhibit different responses under various pressure ranges, with medium pressure promoting advantageous structural modifications for gelation, whereas high pressure results in total denaturation.

As discussed in section 4.2.2, cold plasma treatment can have different effects on protein solubility depending on the treatment time. It was reported that the solubility of sunflower seed protein treated with cold plasma for 3 min increased, whereas treatment for 4 and 5 min decreased the solubility (Wang et al. 2024). However, for soybean protein isolates, cold plasma treatment for more than 10 min increased the solubility by more than 30% (Bu et al. 2022). The notable difference in solubility results could be due to the unique characteristics of each protein source. Sunflower seed protein is reported to be sensitive to prolonged exposure due to its specific amino acid composition and protein structure. This was also supported by (Kehm et al. 2021), who reported sulfur‐rich proteins (e.g., cysteine in milk β‐lactoglobulin) are more prone to oxidation by cold plasma reactive species, leading to disulfide bond formation and aggregation, potentially reducing their solubility during prolonged treatment. This clearly indicates that proteins from different sources exhibit variations in their amino acid sequences and properties, which affects their response to processing. Another possible reason could be the pH at which the solubility of the proteins is measured; that is, the solubilities measured at pH 7 and 12 may differ due to charge‐state changes (Turker and Isleroglu 2024).

A variation in the solubility and degree of hydrolysis of proteins from different foods treated with enzymatic hydrolysis was observed. For shrimp heads, the addition of 3% papain enzyme resulted in the highest degree of protein hydrolysis and increased soluble protein content with increasing papain enzyme concentration (Dzikri Hisyam et al. 2024). However, in chlorella, although all the hydrolysates resulted in more than 50% protein solubility following enzymatic hydrolysis, the trypsin and pepsin hydrolysates showed the highest and lowest degree of hydrolysis, respectively, among the different enzymes tested (Gharehbeglou et al. 2024). The variations in hydrolysis degree and solubility are attributable to the selectivity of enzymes for proteins from different sources. Papain may have a stronger affinity for the proteins in shrimp heads, resulting in more efficient hydrolysis. The various enzymes in chlorella have distinct cleavage specificities, resulting in variable degrees of hydrolysis and solubility.

The enzymatic hydrolysis of soybean meal hydrolysates reduced the particle size, increased the total amino acid content, and improved the emulsification capacity for a short period, but prolonged hydrolysis negatively affected all these protein characteristics (Zhang, Du, et al. 2023). On the other hand, a medium enzymatic hydrolysis treatment time for egg yolk increased the gelling points, improved the thermal stability, and enhanced the emulsifying stability (Gao et al. 2019). According to their study, enzymatic hydrolysis of egg yolk protein increases solubility at pH values away from the isoelectric point due to enhanced electrostatic repulsion, indicating the effect of pH on protein modifications. The complex structure of soybean meal protein requires a specific hydrolysis pattern to improve emulsification, and the unique functional groups of egg yolk protein, which are regarded as suitable for enzymatic hydrolysis, could also explain the variations in functional properties.

Thermally assisted protein modification methods such as ohmic heating and ultrasonic heating show temperature‐dependent effects on food proteins. For example, ohmic heating of sesame protein at 85°C improves protein solubility by unfolding and exposing polar groups, but treatment at high temperature reduces solubility due to cross‐linking (Saeedabad et al. 2024). Although other factors may play a substantial role in the variations in the responses of food proteins to novel food processing, amino acid composition and structure, native protein conformation, treatment intensity and duration, temperature and pH during processing, and measurement techniques and analytical methods are the main factors. To address those kinds of variabilities, future research in this sector must focus on standardized reporting of processing parameters (e.g., voltage gradient in ohmic heating, pulse waveform in PEF, and plasma gas composition in cold plasma) to enable reproducibility, protein source characterization, multi‐method analytical approaches, and statistical modeling of parameter interactions.

## 9 Future Perspectives

### 9.1 Combined Technologies

Although novel food processing methods have interesting effects on various food protein properties, every novel food processing method has its own drawbacks. To reduce their limitations and unfavorable influence on food protein and for better protein modifications, thermal and nonthermal processing methods can be applied. For example, ultrasonic heating can be combined with nonthermal plasma‐activated water by using the latter as a pretreatment for food proteins. This approach can enhance protein structure, increasing susceptibility to ultrasonic heating, which helps avoid the common issue of protein denaturation caused by prolonged ultrasound exposure. Furthermore, ultrasonic heating provides the activation energy needed for the reactive species in both plasma and protein. As a result, this method can save time and energy during the protein modification process.

### 9.2 Simulation Softwares

**FIGURE 8:**
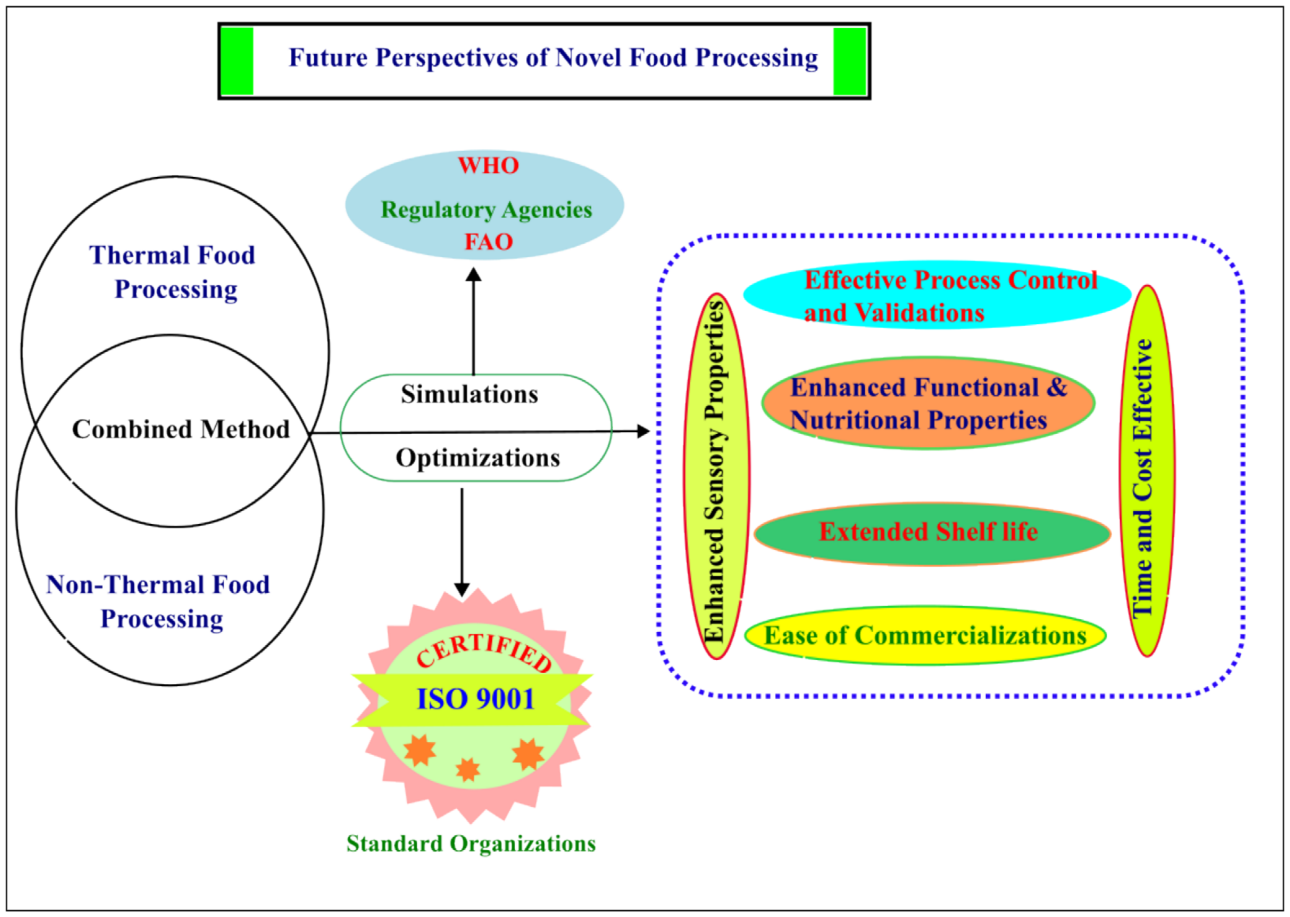
Future perspectives on novel food processing methods for protein modifications.

Computer‐based tools (simulation software) can also be used to predict the interactions between combined food processing methods and food components clearly, thereby resolving the complexity associated with scaling up, controlling processes, and validating novel food processing methods (Figure 8). For example, food researchers can utilize COMSOL Multiphysics to better understand the interactions between different species during plasma processing, a complex operation that is challenging to grasp in practical applications.

Similarly, enzymatic hydrolysis modifications of food proteins face constraints such as high‐energy consumption to maintain reaction conditions, expensive enzymes with limited reusability, significant scalability costs, and strict regulatory requirements. Simulation software such as COMSOL can model and optimize reaction parameters, that is, temperature, pH, and the enzyme‐substrate ratio to save energy; predict enzyme activities for effective utilization and cost saving; and simulate the scale‐up and sensitivity analyses for identifying cost‐effective equipment, utilities, and methodology choices. Furthermore, immobilized enzyme technologies (a vibrant area of research in the fields of biotechnology and chemical engineering) can enhance enzyme reusability while lowering prices, and research into more efficient enzyme production methods can also reduce costs. Transparencies and strong collaborations with regulatory agencies from project start‐ups are essential to ensure compliance regarding food safety and enzyme usage. Other modeling and simulation software, such as ANSYS, could also be employed to predict phenomena during novel food processing of food proteins, facilitating the scaling up and precise control of process parameters.

In addition to the aforementioned novel food processing methods, PEF modifications of food proteins also have limitations related to high‐energy consumption and nonthermal treatment effects, which can lead to inconsistent results and can be mitigated by optimizing the PEF system design, adjusting the solution conditions, and improving the treatment chambers. Though only typical constraints of some of the novel food processing methods in food protein modification are highlighted here, most of them share similar constraints except for their specific operation variations and process parameters; therefore, it is believed that the two proposed perspectives (combined technologies and simulation software) can mitigate their major constraints.

Furthermore, extensive research should be conducted on the optimization of process parameters for combined processing methods, as well as their impact on the particle size, solubility, structure, emulsion and foaming stability, and bioactivity of food proteins, as combined treatments might result in several parameters affecting the modification process. In the future, a combination of thermal and nonthermal food processing, as well as simulations and optimizations, is expected to result in improved sensory properties, effective process control and validation of operations, improved nutritional and functional properties, extended shelf life, ease of commercialization, and time‐ and cost‐efficient operations.

Finally, for a long time, developing clear standards for novel processing methods and ensuring compliance with regulatory agencies has been a challenging trend. However, with the aforementioned perspectives of combined treatments and computer tools, it may be possible to achieve optimal combination effects and establish clear standards, thereby facilitating the approval and certification process.

## 10 Conclusions

Novel food processing technologies, such as ohmic heating, ultrasonic heating, cold plasma, plasma‐activated water, high‐pressure processing, pulsed electric fields, and enzymatic hydrolysis, have evolved to meet the ever‐growing demand of customers for safe, nutritious, and minimally processed foods with extended shelf life. In this review, the impacts of the aforementioned novel food processing techniques on the structure, particle size, solubility, emulsion and foaming stability, and bioactivity of food proteins, as well as their overall impact on the functional and nutritional properties of foods, are discussed. According to the literature, ohmic heating provides rapid and uniform heating and tends to affect protein quality in different foods, that is, meat, milk, and bread. Ultrasound heating, a nonthermal process, enhances the physicochemical and functional properties of food proteins, such as their water holding and foaming capacities. HPP is reported mainly to affect the particle size, secondary structure, and coagulation properties of food proteins. PEF, which is well known for its low‐temperature and short‐processing time requirements, has been shown to improve protein solubility and modify protein structure, thereby enhancing the apparent viscosity of food products. Enzymatic hydrolysis breaks down food macromolecules, improving texture, proteolytic activity, nutrition, and flavor. It also increases the degree of protein hydrolysis and solubility, modifying protein functional characteristics.

Overall, these novel food‐processing methods have diverse effects on food proteins, which might be positive or negative depending on the processing conditions and the source of the protein. Understanding the overall effects from nutritional, quality, and engineering perspectives will facilitate process parameter optimization, enhance food quality, and aid in the development of innovative foods with protein as the main ingredient. Therefore, to bridge the existing knowledge gaps and drive advancements in this hot sector, future research should focus on a clear understanding of the mechanisms of these effects, optimization of process parameters for best protein effects, and widening the potential applications of modified food protein in the food industry.

## Author Contributions

**Shuai Wei:** conceptualization (lead), investigation (equal), writing – original draft (equal), writing – review and editing (equal). **Anand Kumar:** formal analysis (equal), methodology (equal). **Gebremichael Gebremedhin Hailu:** writing – original draft (equal), writing – review and editing (equal). **Sun‐Il Choi:** formal analysis (equal), writing – original draft (equal). **Ok‐Hwan Lee:** formal analysis (equal), writing – original draft (equal). **Ramachandran Chelliah:** writing – review and editing (equal). **Deog‐Hwan Oh:** writing – review and editing (equal). **Shucheng Liu:** investigation (equal), writing – review and editing (equal).

## Conflicts of Interest

The authors declare no conflicts of interest.

## Data Availability

Data sharing is not applicable to this article, as no datasets were generated or analyzed during the current study.
